# TFE3 Regulates the Function of the Autophagy-Lysosome Pathway to Drive the Invasion and Metastasis of Papillary Thyroid Carcinoma

**DOI:** 10.1155/2021/3081491

**Published:** 2021-10-07

**Authors:** Hongsheng Lu, Chumeng Zhu, Yanyun Ruan, Lilong Fan, Kena Wei, Zhaohui Yang, Qi Chen

**Affiliations:** ^1^Department of Pathology, Taizhou Central Hospital (Taizhou University Hospital), Taizhou, Zhejiang 318000, China; ^2^Precision Medicine Center, Taizhou Central Hospital (Taizhou University Hospital), Taizhou, Zhejiang 318000, China

## Abstract

**Background:**

Accumulating evidence shows that autophagy plays a vital role in tumor occurrence, development, and metastasis and even determines tumor prognosis. However, little is known about its role in papillary thyroid carcinoma (PTC) or the potentially oncogenic role of TFE3 in regulating the autophagy-lysosome system.

**Methods:**

Immunohistochemistry and quantitative real-time PCR (qRT-PCR) were used to examine the expression of TFE3, P62/SQSTM1, and LC3 in PTC and paracancerous tissues. TFE3, P62/SQSTM1, LC3, cathepsin L (CTSL), and cathepsin B (CTSB) were evaluated using Western blot analysis. After inducing TFE3 overexpression by plasmid or TFE3 downregulation by small interfering RNA (siRNA) transfection, MTT, wound healing, and cell migration and invasion assays were used to verify the effects on invasion, migration, and the levels of autophagy-lysosome system-related proteins such as P62/SQSTM1, LC3, CTSL, and CTSB.

**Results:**

TFE3 was overexpressed in PTC tissues compared with paracancerous tissues. Analysis of the clinicopathological characteristics of PTC patients showed that high TFE3 expression was significantly correlated with lymph node metastasis. TFE3 overexpression in the PTC cell lines KTC-1 and BCPAP promoted proliferation, invasion, and migration, while TFE3 knockdown had the opposite effects. Furthermore, we identified a positive relationship among the expression levels of TFE3, P62/SQSTM1, LC3, CTSL, and CTSB. We found that silencing TFE3 inhibited the expression of P62/SQSTM1, LC3, CTSL, and CTSB in PTC cells. However, TFE3 overexpression had the opposite effects.

**Conclusions:**

The present study provided evidence for the underlying mechanisms by which TFE3 induces autophagy-lysosome system activity in PTC.

## 1. Introduction

Thyroid cancer (TC) is the most common endocrine malignancy [1], and its incidence has increased rapidly over the past decades [2]. Thyroid cancer can be classified as follicular thyroid cancer (FTC), papillary thyroid carcinoma (PTC), medullary thyroid carcinoma (MTC), or anaplastic thyroid carcinoma (ATC), and PTC accounts for approximately 80% of all thyroid carcinomas [3]. Although the prognosis of PTC is better than that of most other tumors, the prognosis of patients with tumors that are insensitive to traditional surgery and iodine chemotherapy is poor. Therefore, exploring new biomarkers is highly important for improving the diagnosis and treatment of PTC.

Transcription factor E3 (TFE3) is located on the short arm of X chromosome 11.22 and is a microphthalmia family member [4]. Recently, the MiT/TFE family was identified as a regulator of autophagy. Subsequent studies showed that TFE3 can bind to CLEAR elements, which are present in the promoter regions of many lysosomal genes, and regulate lysosomal biogenesis in several different cell types [5].

Autophagy is a highly evolutionarily conserved catabolic process essential for the maintenance of cell homeostasis and adaptation to various stress conditions [6]. Autophagy is primarily a response to microenvironmental stressors, such as hypoxia, nutrient deficiency, and the accumulation of reactive oxygen species (ROS). Dysfunctional autophagy contributes to many diseases, including cancer. Depending on the type and stage of the cancer, autophagy can play a positive or negative role in cancer development [7]. Thus, understanding the role of autophagy in PTC is crucial for identifying new therapeutic targets for PT. Gene set enrichment analysis (GSEA) was used to analyze the enrichment of autophagy- and lysosome-related biological functions in PTC. The expression of genes involved in autophagy and lysosome-related biological functions was upregulated in the PTC group.

In the present study, we investigated the expression of the autophagy regulator TFE3 in PTC and clarified its role as a potential target in PTC patients.

## 2. Material and Methods

### 2.1. Tissue Samples

Seventy-eight pairs of PTC tissues and paracancerous tissues were acquired from patients who underwent surgical resection at Taizhou Central Hospital between March 2017 and January 2019. Patients with other tumors or a history of other tumors were excluded from this study. The patients were not given any chemotherapy or radiotherapy. The PTC population comprised 56 women and 22 men ranging in age from 18-79 years. All tissue samples were snap frozen in liquid nitrogen and quickly stored at -80°C. The clinicopathological characteristics of the PTC patients are listed in Table 1. The clinical stage, tumor stage, and lymph node stage of the patients are listed according to the tumor-node-metastasis (TNM) classification [8]. In addition, this research was approved by the Ethics Committee of Taizhou Central Hospital (Taizhou, China). Formal written approval was obtained from each patient.

### 2.2. Cell Lines and Cell Culture

Two PTC cell lines (KTC-1 and BCPAP) and a human thyroid follicular cell line (Nthy-ori 3-1) were obtained from the Cell Bank of the Chinese Academy of Sciences (Shanghai, China). All cells were cultured in RPMI 1640 medium (REF: 61870036, Gibco, USA) supplemented with 10% fetal bovine serum (REF: 10270106, Gibco, USA), 1% GlutaMAX (REF: 35050061, Gibco, USA),1% MEM nonessential amino acid solution (REF: M7145, Sigma, USA), and 1% sodium pyruvate (100 mM) solution (REF:11360070, Gibco, USA), hereafter referred to as complete culture medium. Cells were incubated at 37°C in a humidified atmosphere with 5% CO_2_ and were passaged routinely.

### 2.3. Cell Transfection

TFE3 was overexpressed by transfection with pcDNA3.1-TFE3. cDNA encoding full-length TFE3 was cloned into the pcDNA vector, and the sequence was confirmed by Sanger sequencing. A small interfering RNA (siRNA) targeting TFE3 (si-TFE3) and the corresponding negative control siRNA (si-NC) were used for TFE3 knockdown. The siRNA sequences were as follows: 5′-GCAGCUCCGAAUUCAGGAACUTT-3′ (sense) and 5′-AGUUCCUGAAUUCGG AGCUGCTT-3′ (antisense).

pcDNA3.1-TFE3 or si-TFE3 was transfected into PTC cells using Lipofectamine® 2000 (REF: 11668019, Invitrogen, Carlsbad, CA, USA) according to the manufacturer's protocol.

### 2.4. Quantitative Real-Time Polymerase Chain Reaction (qRT-PCR)

Total RNA was extracted with TRIzol reagent (REF: 15596018, Invitrogen, USA) and reverse transcribed into cDNA with an RT Kit (REF: K1622, Applied Biosystems, USA) before qRT-PCR. qRT-PCR was carried out using SYBR® qPCR Mix (REF: A25742, Applied Biosystems, USA) in an ABI 7500 Fast Real-Time PCR analysis system (Applied Biosystems, USA). The sequences of all primers used for PCR are listed as follows: TFE3 (forward): 5′-ACTGGGCACTCTCATC CCTAAGTC-3′, TFE3 (reverse): 5′-TTCAGGATGGTG CCCTTGTTC-3′, P62/SQSTM1 (forward): 5′-ATCGGAGGATCCGAGTGT-3′, P62/SQSTM1 (reverse): 5′-TGGCTGTGAGCTGCTCTT-3′, LC3 (forward): 5′-ACCATGCCGTCGGAGAAG-3′, LC3 (reverse): 5′-ATCGTTCTATTATCACCGGGATTTT-3′, *β*-actin (forward): 5′-TGACGTG GACATCCGCAAAG-3′, and *β*-actin (reverse): 5′-CTGGAAG GTGGACAGCGAGG-3′.

*β*-Actin was used as the internal control. The relative quantification of each sample was performed using the 2^−ΔΔCt^ method.

### 2.5. Wound Healing Assay

Cells were cultured in 6-well plates at a density of 80%, and a 200 *μ*l pipette tip was then used to create scratches on the surface of the cultured cells. Cells were washed three times with phosphate-buffered saline (PBS) and cultured in serum-free RPMI 1640 medium. Wounds were observed under a microscope, and images were acquired at 0 h and 24 h. The results were analyzed with ImageJ software (National Institutes of Health, Bethesda, MD, USA), and the rate of cell migration was determined as follows: ((diameter of original wound − diameter of wound at different time points)/diameter of original wound) × 100%.

### 2.6. Cell Migration and Invasion Assays

Cell invasion was performed using a Transwell assay. In brief, PTC cells were trypsinized, suspended in serum-free RPMI 1640 medium (100 *μ*l), and seeded in the upper chamber of each Matrigel-coated Transwell insert (REF: 3422, Corning, USA). Complete culture medium (500 *μ*l) was added to the lower chambers. After 24 h of incubation, cells in the upper chamber had traversed to the bottom surface of the membrane, which was fixed with methanol and then stained with 0.1% crystal violet. The migration assay was conducted with the same procedure used for the invasion assay except that the membrane was not coated with Matrigel. Random fields were selected for imaging with a Zeiss photomicroscope (Carl Zeiss Meditec, Dublin, CA, USA).

### 2.7. MTT Assay

Cell proliferation was evaluated by using a 3-[4,5-dimethylthiazol-2-yl]-2,5-diphenyltetrazolium bromide (MTT) assay. KTC-1 and BCPAP cells (3 × 10^3^ cells/well) transfected for 24 h were cultured in 96-well plates for 0, 24, 48, or 72 h. Then, 0.1 mg/ml MTT (REF: M818, Solarbio, China) (50 *μ*l) was added to each well and incubated at 37°C. After 4 h, 150 *μ*l of DMSO (REF: D8371, Solarbio, China) was added and incubated with gentle shaking at room temperature for 1 h. Cell viability was assessed by measuring the absorbance with a microplate reader at 570 nm.

### 2.8. Western Blot Analysis

Total cellular protein was extracted with RIPA lysis buffer (REF: 78501, Thermo, USA) and protease inhibitors, diluted to equal concentrations with 5× loading buffer, and denatured at 95°C. Then, 20 *μ*g of denatured protein was subjected to sodium dodecyl sulfate (SDS)-polyacrylamide gel electrophoresis. Proteins were electrotransferred to nitrocellulose (NC) membranes, blocked with 5% bovine serum albumin (BSA, REF: A8020, Solarbio, China) at room temperature for 1 h, and incubated with primary antibodies against TFE3 (1 : 1000, REF: 14779S, Cell Signaling Technology, USA), P62/SQSTM1 (1 : 1000, REF: 184201AP, Proteintech, USA), LC3 (1 : 2000, REF: L7543, Sigma-Aldrich, USA), cathepsin B (CTSB, 1 : 1500; REF: AF5189, Affinity, China), and cathepsin L (CTSL, 1 : 1000, REF: DF12880, Affinity, China) at 4°C overnight. The membranes were washed three times with PBS containing Tween 20 (PBST), incubated with anti-rabbit (REF: 31460) or anti-mouse (REF: 31430) secondary antibodies (1 : 2000; Pierce, Appleton, WI, USA) at room temperature for 1 h, and washed three more times with PBST. Protein bands were visualized using enhanced chemiluminescence reagent (REF: 32109, Pierce, Appleton, WI, USA). *β*-Actin (1 : 1000, REF: 3700, Cell Signaling Technology, USA) was used as the internal reference, and the relative protein expression levels were determined based on calculation of the target band/internal control band gray value ratios.

### 2.9. Immunohistochemistry

Tissue sections were deparaffinized with xylene and then hydrated with alcohol, and antigen retrieval was performed by heating in 0.01 sodium citrate buffer in a microwave oven for 15 minutes. The sections were incubated with 3% hydrogen peroxide for 10 minutes to block endogenous peroxidase activity. Nonspecific binding was prevented by adding normal goat serum for blocking for 20 minutes. Sections were incubated first with primary antibodies against TFE3 (1 : 500; Cell Signaling Technology, USA), P62/SQSTM1 (1 : 50; Proteintech, USA), and LC3 (1 : 500; Sigma-Aldrich, USA) for 1 h at room temperature and then with anti-rabbit or anti-mouse secondary antibodies (1 : 500; Pierce, Appleton, WI, USA) at room temperature for 1 h. After each treatment, sections were washed 3 times with TBST for 5 minutes each, and color was then developed with 3,3′-diaminobenzidine. Sections were counterstained with hematoxylin, differentiated with a hydrochloric acid alcohol solution, dehydrated, cleared, and fixed. Random fields were selected for imaging with a Zeiss photomicroscope (Carl Zeiss Meditec, Dublin, CA, USA).

### 2.10. Statistical Analysis

All experimental procedures were repeated at least three times. Data were analyzed using SPSS 20.0 software (SPSS Inc., Chicago, IL, USA) and GraphPad Prism 8.0 statistical software (GraphPad Software Inc., La Jolla, CA, USA). Associations between expression levels were analyzed with Spearman correlation analysis. The Mann–Whitney *U* test was used to compare the mean values of more than two groups. All data are expressed as the mean ± standard deviation (SD) values, and a *P* value < 0.05 indicates statistical significance.

## 3. Results

### 3.1. Autophagy-Lysosome System Activity Is Positively Correlated with PTC Progression

**Figure 1:**
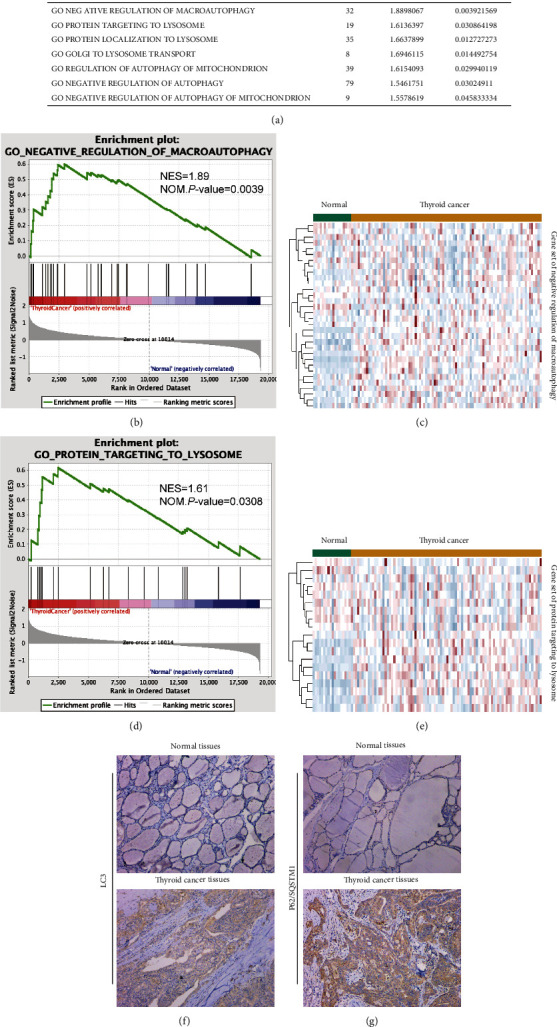
Autophagy was positively correlated with thyroid cancer progression. Gene set enrichment analysis (GSEA) showed that the expression of genes involved in autophagy and lysosome-related biological functions was upregulated in thyroid cancer. (a) GSEA of the data revealed that human thyroid cancer specimens have elevated expression of autophagy-lysosome genes compared with normal thyroid tissue. NES: normalized enrichment score; Nom.: nominal. (b) GSEA showing the correlation between thyroid cancer and the macroautophagy gene signature. (c) Upregulation of macroautophagy genes in thyroid cancer relative to matched normal tissue. (d) GSEA showing the correlation between thyroid cancer and the lysosome gene signature. (e) Upregulation of lysosome genes in thyroid cancer relative to matched normal tissue. (f, g) Immunohistochemistry assays showed an increase in P62/SQSTM1 and LC3 expression in thyroid cancer tissues compared with normal tissues.

To explore the role of the autophagy-lysosome system in the process of PTC, GSEA was used to analyze the enrichment of autophagy- and lysosome-related biological functions in PT. The expression of genes involved in autophagy- and lysosome-related biological functions was upregulated in the PTC group, and the functional enrichment analysis showed significant differences (Figures 1(a)–1(e), *P* < 0.05). Consistent with the above results, the results of immunohistochemical assays indicated that the expression levels of LC3 and P62/SQSTM1 in PTC tissues were significantly higher than those in paracancerous tissues (Figures 1(f) and 1(g)).

### 3.2. TFE3 Is Overexpressed in PTC Tissues and Cells

**Figure 2:**
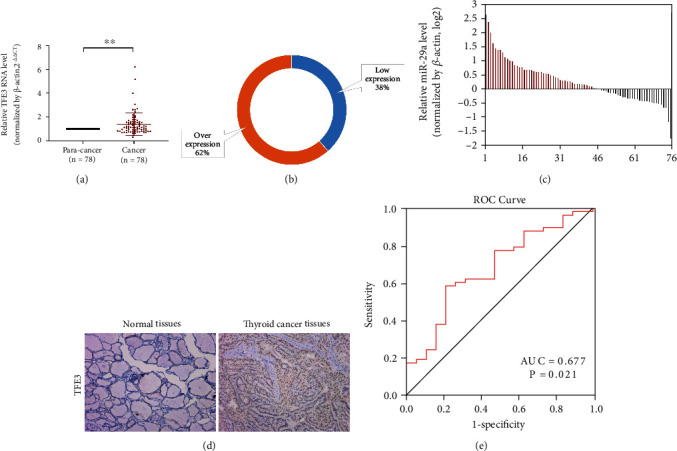
TFE3 was overexpressed and closely associated with lymph node metastasis in PTC. (a–c) TFE3 mRNA expression was significantly higher in PTC than in normal thyroid tissues. (d) Immunohistochemistry assays showed an increase in TFE3 gene expression in PTC tissues compared with normal tissues (^∗∗^*P* < 0.01). (e) The ROC curve of TFE3 for lymph node metastasis in PTC.

**Figure 3:**
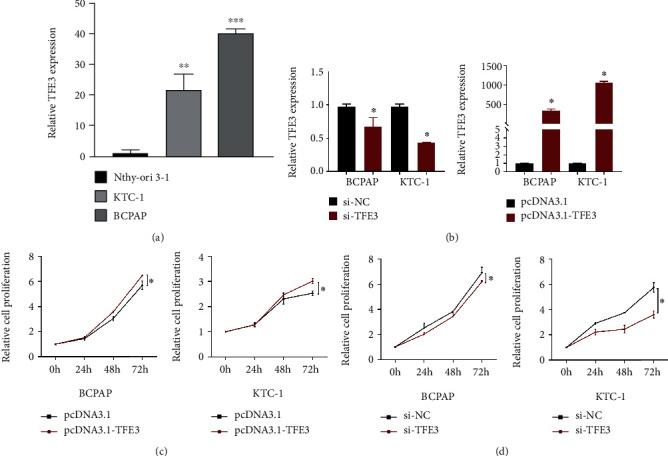
TFE3 promotes proliferation of PTC cells. (a) The expression of TFE3 in PTC cells was higher than that in Nthy-ori 3-1 (^∗∗^*P* < 0.01 and ^∗∗∗^*P* < 0.001). (b) After TFE3 transfection, the expression of TFE3 was detected by real-time PCR. (c) MTT assay showed that overexpression of TFE3 significantly increased the proliferation of BCPAP and KTC-1 cells (^∗^*P* < 0.05). (d) MTT assay showed that knockdown of TFE3 decreased the proliferation of BCPAP and KTC-1 cells (^∗^*P* < 0.05).

We examined the level of TFE3 in 78 pairs of PTC tissues and paracancerous tissues. The qRT-PCR results showed that TFE3 expression was significantly increased in PTC tissues compared with paracancerous tissues (Figures 2(a)–2(c), *P* < 0.01). Then, we analyzed the correlations between clinicopathological characteristics and TFE3 expression in PTC patients. The results indicated that high expression of TFE3 was significantly correlated with lymph node metastasis (Table 1, Figure 2(e), *P* < 0.05). Moreover, the results of immunohistochemical assays confirmed that the expression of TFE3 in PTC tissues was higher than that in paracancerous tissues (Figure 2(d)). We examined the level of TFE3 in Nthy-ori 3-1, KTC-1, and BCPAP. The qRT-PCR results showed that TFE3 expression was significantly increased in KTC-1 and BCPAP compared with Nthy-ori 3-1 cells (Figure 3(a), *P* < 0.01).

### 3.3. TFE3 Promotes the Proliferation of PTC Cells

Here, we detected the effect of differential TFE3 expression on PTC cell proliferation through an MTT assay. As shown in Figure 3(d), downregulation of TFE3 expression significantly inhibited cell growth compared with that of si-NC-transfected cells. Consistent with the results in the si-TFE3 group, the pcDNA3.1-TFE3 group demonstrated a much higher cell proliferation ability than the pcDNA3.1 group (Figure 3(c), *P* < 0.05). These data suggested that TFE3 promoted the proliferation of PTC cells. The assays were repeated in triplicate.

### 3.4. TFE3 Accelerates PTC Cell Migration and Invasion In Vitro

**Figure 4:**
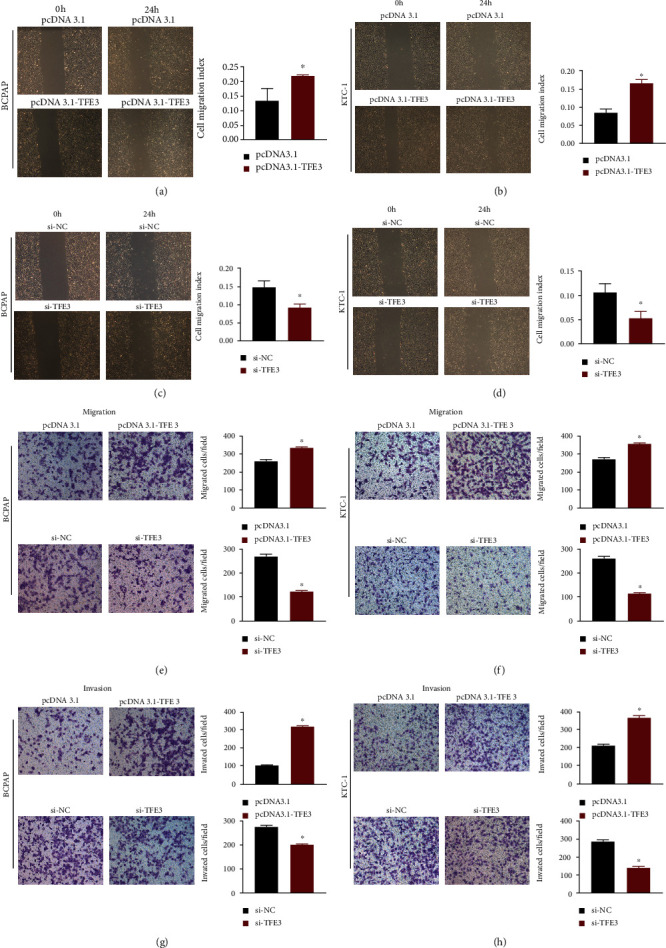
TFE3 accelerates PTC cell migration and invasion in vitro. (a, b) Wound healing assays showed that overexpression of TFE3 increased thyroid cancer cell migration. (c, d) Silencing of TFE3 reduced PTC cell migration. (e–h) Transwell assays indicated that overexpression of TFE3 increased the migratory and invasive ability of PTC cells, while knockdown of TFE3 decreased the migratory and invasive ability of PTC cells (^∗^*P* < 0.05).

To determine the role of TFE3 in PTC progression, we conducted wound healing and Transwell assays to examine its effects on migration and invasion. PTC cells were transfected with pcDNA3.1-TFE3 or si-TFE3 in 6-well plates. First, the results of the wound healing assay revealed that TFE3 transfection increased the migration ability of PTC cells (Figures 4(a)–4(d), *P* < 0.05). In addition, the Transwell assay results showed that the migration of PTC cells was significantly suppressed in the si-TFE3 group compared with the si-NC group, while more cells penetrated the lower surface of the membrane in the pcDNA3.1-TFE3 group than in the pcDNA3.1 group (Figures 4(e) and 4(f), *P* < 0.05). Consistent with the migration assay results, the invasion assay results showed that cell invasion was noticeably suppressed by TFE3 knockdown and enhanced by TFE3 overexpression (Figures 4(g) and 4(h), *P* < 0.05). In conclusion, these results indicated that TFE3 might promote migratory and invasive behaviors in PTC cells. The assays were performed in triplicate.

### 3.5. TFE3 Induces Autophagy-Lysosome System Activity in PTC Cells

**Figure 5:**
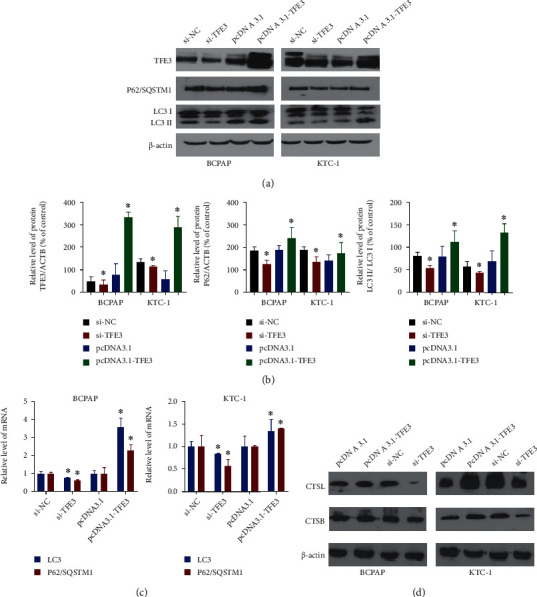
TFE3 induces autophagy-lysosome in PTC cells. (a, b) After transfection with pcDNA3.1-TFE3 or si-TFE3, BCPAP and KTC-1 cells showed significant changes in TFE3 protein levels. Overexpression of TFE3 increased the expression of P62/SQSTM1, and the opposite results were observed in BCPAP and KTC-1 cells transfected with si-TFE3. The ratio of LC3II/LC3Iin BCPAP and KTC-1 cells increased significantly after pcDNA3.1-TFE3 transfection, and the opposite results appeared after si-TFE3 transfection. (c) After knocking down TFE3, the expression of LC3 and P62/SQSTM1 in PTC cells was decreased, and overexpression of TFE3 increased the expression of LC3 and P62/SQSTM1 in PTC cells (^∗^*P* < 0.05). (d) After knocking down TFE3, the expression of CTSB and CTSL in PTC cells was decreased, while TFE3 is overexpression, the expression of CTSB and CTSL in PTC cells increased (^∗^*P* < 0.05).

We sought to determine whether autophagy-lysosome system activity is related to TFE3. As shown in Figure 5(c), the expression levels of LC3 and P62/SQSTM1 in the pcDNA3.1-TFE3 group were higher than those in the pcDNA3.1 group. Then, we detected the conversion of LC3 I to LC3 II, which is an indicator of autophagy, in BCPAP and KTC-1 cells transfected with pcDNA3.1-TFE3 or si-TFE3 [9]. TFE3 increased the LC3 (Figures 5(a) and 5(b), *P* < 0.05). Next, we examined the protein level of P62/SQSTM1. P62/SQSTM1 is selectively incorporated into autophagosomes by simultaneously interacting with the LC3 protein [10]. Compared with the pcDNA3.1 group, the pcDNA3.1-TFE3 group showed increased expression of P62/SQSTM1 (Figures 5(a) and 5(b)). Similarly, compared to the si-NC group, the si-TFE3 group showed decreased P62/SQSTM1 expression. Through qRT-PCR experiments, we found that the mRNA expression levels of LC3 and P62/SQSTM1 in PTC cells were decreased after TFE3 knockdown and increased after TFE3 overexpression (Figure 5(c), *P* < 0.05). To better explore autophagy in PTC cells, we measured the activity of lysosomal enzymes (CTSB and CTSL). Compared with the pcDNA3.1 group, the pcDNA3.1-TFE3 group showed increased activity of CTSB and CTSL (Figure 5(d)). Similarly, the activity of CTSB and CTSL in the si-NC group was higher than that in the si-TFE3 group (Figure 5(d)). The above results revealed that TFE3 might be able to promote autophagy-lysosome system activity in PTC cells. The assays were performed in triplicate.

## 4. Discussion

PTC is the most common endocrine malignancy and is the 5th most common female tumor [2]. Currently, PTC treatment is based on ^131^I radiotherapy and chemotherapy combined with thyroid-stimulating hormone (TSH) suppression therapy after surgical resection [11]. However, tumor recurrence and metastasis and the presence of chemotherapeutic resistance in refractory PTC lead to decreased survival rates of patients with PTC [12].

TFE3, which belongs to the MiT/TFE family, is a regulator of autophagy and lysosomal biogenesis [13]. In 2009, the MiT/TFE family was initially discovered to be able to regulate most lysosomal genes (including promoters encoding hydrolases and lysosomal proteins) [14]. Importantly, under stress conditions, complex interactions between MiT/TFE family-dependent autophagic homeostasis pathways and apoptotic processes may occur in cancer cells, and these interactions ultimately determine the fate of these cells in relation to cell death or survival [15]. TFE3 simultaneously regulates autophagy induction, lysosomal biogenesis, oxidative metabolism, and oxidative stress and thus plays an important role in determining cell fate [16]. Notably, under nonstress conditions, TFE3 interacts with 110 14-3-3 proteins, remains in the cytoplasm, and is phosphorylated at Ser321. In contrast, under stress conditions, TFE3 is dephosphorylated, the TFE3/14-3-3 complex dissociates, and TFE3 translocates from the cytoplasm to the nucleus to promote autophagy and lysosome biogenesis [5]. The TFE3 gene was found to be fused with the papillary renal cell carcinoma (PRCC) gene on chromosome 1q21.2 [PRCC-TFE3 t(X;1)(p11.2;q21)] [17]. Moreover, Fan et al. [18] found that inhibiting MT2-TFE3-dependent autophagy enhanced melatonin-induced apoptosis in tongue squamous cell carcinoma. Furthermore, in renal cell carcinoma (RCC), increased TFE3 expression was associated with poor progression-free survival (PFS) [19]. Consistent with these results, we found that TFE3 increased the proliferation and invasion of PTC cells and decreased their apoptosis.

M. Anselmier, a French physiologist, first used the term “autophagy” in a short article published in 1859 describing the effects of fasting on mice [20]. Autophagy, a protein degradation pathway that is highly conserved from yeast to humans, is essential for clearing protein aggregates and misfolded proteins from healthy cells. Under stress conditions, cells produce many damaged proteins or organelles, and double-membrane vesicles reform in the cytoplasm to engulf defective or toxic molecules and organelles to subsequently form autophagosomes. Then, autophagosomes fuse with lysosomes and release lysosomal acid lipase in the vesicles to degrade toxic molecules and other substances, and the resulting products are used to resynthesize new proteins or organelles [21]. The whole process of autophagy involves a variety of evolutionarily conserved genes, namely, autophagy-related genes (ATGs) [22]. Autophagy can be classified as macroautophagy, microautophagy, and molecular chaperone-mediated autophagy (CMA) according to the different ways of transporting cellular material to lysosomes [23]. Previous studies demonstrated that autophagy is an important participant in the pathogenesis of many diseases, including cancer [24, 25]. In addition, genome-wide association studies showed that ATG5 is associated with systemic lupus erythematosus (SLE) in Chinese individuals, indicating that autophagy may be related to the pathogenesis of SLE [26]. Zou et al. [27] found that suppressing autophagy can enhance the chemotherapeutic effects of paclitaxel in cervical cancer cells. However, the relationship between PTC and autophagy has not been fully elucidated.

In this study, 90 samples of PTC tissue and 18 samples of paracancerous tissue were selected from the TCGA database to analyze the mechanism of PTC. The enrichment of autophagy- and lysosome-related biological functions involved in LC3 and P62/SQSTM1 in PTC data was analyzed by GSEA, which showed that autophagy-lysosome system activity was positively correlated with thyroid cancer progression. LC3 and P62/SQSTM1 have been widely reported as indicators of autophagy [28, 29]. CTSB and CTSL are lysosomal acid cysteine proteases that modulate autophagy processes [30]. In addition, TFE3 was identified as a regulator of autophagy in previous studies [16]. Based on bioinformatic analysis and GSEA data, we further validated this role in tissues and cells in vitro. We found that the TFE3 level was significantly higher in a set of 78 PTC tissues than in the paired paracancerous tissues. High expression levels of TFE3 were closely associated with lymph node metastasis. Then, we conducted functional assays in KTC-1 and BCPAP cells and found that TFE3 enhanced the proliferation, invasion, and migration of PTC cells by regulating the autophagy-lysosome system, suggesting that TFE3 is a potential sensitive marker in PTC. In this study, we found that autophagy was induced by TFE3, as evidenced by the upregulation of P62/SQSTM1 protein expression and the increased LC3II/LC3I ratio. Therefore, we hypothesized that TFE3 positively regulates the autophagy-lysosome system in PTC.

However, the mechanisms underlying TFE3-mediated autophagy-lysosome system activity and PTC remain unclear. Recent studies have linked the accumulation of ROS to TFE3 activation in cancer prognosis [31]. The potential mechanisms underlying the links among autophagy-lysosome system activity, TFE3, and PTC require additional in-depth research. The study showed that the high expression of TFE3 was significantly correlated with lymph node metastasis, which reflected the possible relationship between lymph node metastasis and autophagy-lysosome levels in PTC patients. Whether autophagy-lysosome-related markers can be used as biomarkers for diagnosis of thyroid papillary carcinoma needs further study.

## 5. Conclusions

In summary, we evaluated the expression of TFE3 and its role in malignant characteristics in PTC, showing that TFE3 promotes PTC progression by regulating the autophagy-lysosome system.

## Figures and Tables

**Table 1:** Correlation between TFE3 expression and clinicopathological characteristics of thyroid cancer patients (*n* = 78).

Clinical parameters	Number	TFE3 expression		*P* value
		Low	High	
Age (years)				*P* = 0.173
<45	42	18	24	
≥45	36	21	15	
Gender				*P* = 1.000
Male	22	11	11	
Female	56	28	28	
Clinical stage^a^				*P* = 0.329
I	67	35	32	
II + III	11	4	7	
Tumor stage^a^				*P* = 0.168
T1 + T2	73	38	35	
T3 + T4	5	1	4	
Lymph node metastasis^a^				*P* = 0.038^∗^
N0	20	14	6	
N1a + N1b	58	25	33	

^∗^ *P* < 0.05 was considered significant. Thyroid carcinoma. ^a^According to the AJCC tumor-node-metastasis (TNM) staging system (eighth edition), 2017.

## Data Availability

Data is available on request.

## Abbreviations

FTC: Follicular thyroid cancer
PTC: Papillary thyroid cancer
MTC: Medullary thyroid cancer
ATC: Anaplastic thyroid cancer
TFE3: Recombinant transcription factor binding to IGHM enhancer 3
ROS: Reactive oxygen species
GSEA: Gene set enrichment analysis
TNM: Tumor-node-metastasis classification
TSH: Thyroid-stimulating hormone
PRCC: Papillary renal cell carcinoma
ATGs: Autophagy-related genes
CMA: Chaperone-mediated autophagy
SLE: Systemic lupus erythematosus
CTSB: Cathepsin B
CTSL: Cathepsin L.
